# Macroalgae Compound Characterizations and Their Effect on the Ruminal Microbiome in Supplemented Lambs

**DOI:** 10.3390/vetsci11120653

**Published:** 2024-12-14

**Authors:** Adriana Guadalupe De la Cruz Gómez, Huitzimengari Campos-García, German D. Mendoza, Juan Carlos García-López, Gregorio Álvarez-Fuentes, Pedro A. Hernández-García, José Alejandro Roque Jiménez, Oswaldo Cifuentes-Lopez, Alejandro E Relling, Héctor A. Lee-Rangel

**Affiliations:** 1 Facultad de Agronomía y Veterinaria, Centro de Biociencias, Instituto de Investigaciones en Zonas Desérticas, Universidad Autónoma de San Luis Potosí, San Luis Potosí 78321, Mexico; adriana.delacruz@uaslp.mx (A.G.D.l.C.G.); jcgarcia@uaslp.mx (J.C.G.-L.); gregorio.alvarez@uaslp.mx (G.Á.-F.); ruben.cifuentes@uaslp.mx (O.C.-L.); 2 Facultad de Medicina Veterinaria y Zootecnia, Benemérita Universidad Autónoma de Puebla, Tecamachalco 75460, Mexico; huitzi.campos@correo.buap.mx; 3 Departamento de Producción Agrícola y Animal, Universidad Autónoma Metropolitana—Xochimilco, Mexico City 04960, Mexico; gmendoza@correo.xoc.uam.mx; 4 Centro Universitario Amecameca, Universidad Autónoma del Estado de México, Amecameca 56900, Mexico; pedro_abel@yahoo.com; 5 Instituto de Ciencias Agrícolas, Universidad Autónoma de Baja California, Ejido Nuevo León, Mexicali 21705, Mexico; jose.roque@uabc.edu.mx; 6 Department of Animal Sciences, The Ohio State University, College of Food, Agricultural, and Environmental Sciences, Wooster, OH 44691, USA; relling.1@osu.edu

**Keywords:** *Macrocystis pyrifera*, *Ulva* spp., *Mazzaella* spp., methanogenesis, rumen

## Abstract

The impact of macroalgae species on rumen function remains largely unexplored. This present study aimed to identify the biocompounds of the three types of marine macroalgae described: *Macrocystis pyrifera* (Brown), *Ulva* spp. (Lettuce), *Mazzaella* spp. (Red) and their effect on species-specific modulations of the rumen microbiome. The macroalgae were characterized using GC-MS. Twelve Rambouillet lambs were randomly assigned to one of four experimental diets (*n* = 3 per treatment): (a) control diet (CD); (b) CD + 5 g of Red algae; (c) CD + 5 g of Brown algae; and (d) CD + 5 g of Lettuce algae. After the lambs ended their fattening phase, they donated ruminal fluid for DNA extraction and 16S rRNA gene V3 amplicon sequencing. Results: The tagged 16S rRNA amplicon sequencing and statistical analysis revealed that the dominant ruminal bacteria shared by all four sample groups belonged to phyla *Firmicutes* and *Bacteroidota*. However, the relative abundance of these bacterial groups was markedly affected by diet composition. In animals fed with macroalgae, the fibrinolytic and cellulolytic bacteria *Selenomonas* was found in the highest abundance. The diversity in chemical composition among macroalgae species introduces a range of bioactive compounds, particularly VOCs like anethole, beta-himachalene, and 4-ethylphenol, which demonstrate antimicrobial and fermentation-modulating properties.

## Simple Summary

Mexico ranked among the ten countries with the highest GHG production in 2017, reporting a contribution of 1.68% of global emissions; in this sense, it has committed to reducing GHG emissions by up to 22% by 2030 (Government of Mexico, 2015). The First National Tier 2 Inventory of methane emissions from enteric fermentation of cattle in Mexico, registering 2039.21 ± 205.5 Gg of CH4 per year. Currently, most studies on livestock and climate change in Latin America are focused on quantifying CH4 emission volumes, determining emission factors, and calculating national inventories; few studies focus on the development of mitigation strategies; many positive attributes of macroalgae have been identified concerning contributing nutrients such as protein and also in the energy metabolism of animals of livestock interest. Some in vitro studies have shown that red and brown macroalgae can reduce CH4 production by controlling the populations of methanogenic bacteria in the rumen.

## 1. Introduction

Macroalgae are sources of minerals, vitamins, proteins, polysaccharides, and bioactive compounds [1]. Thus, macroalgae have been proposed for human and animal nutrition as a healthcare solution [2]. Nevertheless, although humans and non-ruminants use macroalgae as a source of carbohydrates, ruminants seem to be the most suitable animals for using macroalgae in their diets because they can use a significant fraction of carbohydrates and cell-wall-bound proteins [1].

The sustainability of livestock production has become a key focus in research, driven by the increasing global demand for food [3]. In the northeast of México, macroalgae present a largely untapped resource that could meet these demands, contingent on the sustainability of their production processes [4]. The macroalgae Brown, Lettuce, and Red are species-integrated multitrophic aquaculture systems in the Gulf of California, representing a marketable product with minimal or no extra input costs while enhancing the environmental sustainability of fish farming [1]. Consequently, these macroalgae are emerging as viable alternatives to conventional plants, although their nutritional value can fluctuate based on species, seasonality, habitat, and external environmental factors [5]. Moreover, the impact of incorporating various macroalgae species into livestock diets on rumen function remains largely unexplored [1].

The fermentation process in the rumen depends on the interplay between various microbial groups, including bacteria, archaea, protozoa, and fungi [6]. The rumen microbiota is crucial in converting dietary components like cellulose, hemicellulose, starch, and protein into microbial proteins and volatile fatty acids, which provide around 70% of the host animal’s daily protein and energy requirements [7]. Consequently, any changes in the rumen microbiota and fermentation process can significantly affect the host animal’s physiology and efficiency [6,8]. Bacteria comprise most of the microbial communities in the rumen, representing approximately 88% of the microbial population, with densities exceeding 10^10^ cells per milliliter [9]. The animal’s diet and species primarily influence the composition of the rumen microbial groups [6]. Understanding how the rumen microbial ecosystem varies across different animal species and dietary supplements makes it possible to develop effective feeding strategies that improve rumen microbial fermentation, including fiber digestion [6,9]. Makkar et al. [10] highlighted several beneficial aspects of macroalgae, particularly in terms of their nutrient contributions, such as protein, and their impact on the energy metabolism of livestock animals. A notable aspect is the presence of bioactive compounds, which may enhance both animal production and health [3]. Ongoing research is exploring the effects of algae as a supplement in ruminant diets, with some studies indicating that algae supplementation improves the mineral status of animals, particularly concerning iodine and selenium [11]. Additionally, specific in vitro studies have demonstrated that red and brown macroalgae can lower methane (CH_4_) emissions, though freshwater green algae appear to have a minimal effect [12], representing a critical step toward achieving sustainable agriculture and addressing global environmental challenges. However, these studies face challenges related to the dosages and the lack of information on the specific bioactive compounds in the algae and the microbiome generated in the rumen [7].

The Brown (*Macrocystis pyrifera*), Lettuce (*Ulva* spp.), and Red (*Mazzaella* spp.) are lesser-known macroalgae worldwide [13,14]. Min et al. [13] described the potential role of Brown, Lettuce, and Red in ruminants through incubation and gas production techniques. The authors concluded that Lettuce and Red algae substantially decreased carbon dioxide emissions. Also, Min et al. [13] mentioned that brown seaweed, such as Macrocystis macroalgae, is effective in the short term in reducing CH_4_ production. Nevertheless, the long-term efficacy is still unknown. The other seaweeds, *Ulva* spp. and *Mazzaella* spp., have been short-characterized; however, diverse in vitro and methanogenesis reports suggest that they have the potential to modulate the bacterial communities and microbial ecosystem [13,14,15].

Based on previous findings, we hypothesized that bioactive compounds in macroalgae could be used as a supplement in ruminants and to modify the ruminal microbiome positively by promoting the activities of rumen microbiota. Thus, the objective of the current study was to identify the biocompounds of the three types of marine macroalgae previously described: *Macrocystis pyrifera*, *Ulva* spp., and *Mazzaella* spp. and their effect on species-specific modulations of the rumen microbiome.

## 2. Materials and Methods

### 2.1. Ethics

The procedures undertaken in this study were approved by the Animal Care and Use Committee of the Veterinary and Animal Science Faculties at the Autonomous University of San Luis Potosí, under protocol number 004/25022015. These procedures adhered to the guidelines established by the Animal Protection Law and Sanitary Regulations of the State of San Luis Potosí, Mexico. All aspects of animal management followed the Federal guidelines outlined in various Mexican Government regulations, including NOM-051-ZOO-1995 for the humane treatment of animals during transportation [16], NOM-062-ZOO-1995 for technical specifications regarding the care and use of animals in multiple settings, such as laboratories, livestock farms, and zoos [17], and NOM-033-ZOO-1995 for humane treatment and animal protection during slaughter [18]. The experiment was conducted at the Agronomy and Veterinary Faculty of the Autonomous University of San Luis Potosí Research Experimental Station in Mexico.

### 2.2. Chemical Characterization of Compounds Presents in Brown, Red, and Lettuce Algae

The macroalgae Brown, Red, and Lettuce were characterized using gas chromatography (GC-HP 6890) coupled with mass spectrometry (MSHP 5973) employing a capillary column measuring 60 m in length, 0.255 mm in diameter, and coated with a 0.25 micrometer film thickness (HP 5MS, Agilent, Santa Clara, CA, USA). The temperature regimen involved an initial 2 min hold at 70 °C, followed by a ramp to 250 °C at a rate of 20 °C/min, then to 290 °C at 5 °C/min, further raised to 300 °C at 1 °C/min, and then to 310 °C at 5 °C/min, maintaining this temperature for 36 min. The injector was set at 250 °C in spitless mode, and helium flowed at a 1 mL/min rate. The mass spectrometry operated in SCAN mode (50–500 *m*/*z*) for compound identification.

### 2.3. Animals and Diets

Twelve Rambouillet male lambs, 40 Kg average weight, were randomly assigned to one of four experimental diets (*n* = 3 per treatment) as follows: (a) the control diet (CD; without algae) consisted of a commercial mixed diet formulated based on the requirements for the age and weight of the lambs in the final period according to the *Nutrient Requirements of Small Ruminants* guidelines [19]; (b) CD + 5 g of Red algae (BajaBrown^®^, Ensenada Baja California, Mexico); (c) CD + 5 g of Brown algae (BajaBrown^®^, Mexico); and (d) CD + 5 g of Lettuce algae (BajaBrown^®^, Ensenada Baja California, Mexico). The Macroalgaes were added daily to individual feeders on top of the diet, with the dosage determined based on previous studies [10,20]. Based on a chemical report about the same macroalgae [20], the crude protein in each macroalga is 10.4% for *Macrocystis pyrifera*, 6.6% for *Ulva* spp., and 10.5% for *Mazzaella* spp.; hence 5 g did not provide a protein intake impact. The diets were provided as total mixed rations, and the lambs were housed in individual pens equipped with feed and water bowls. According to the manufacturer (Vali Commercial^®^, San Luis Potosi Mexico), the chemical composition of the commercial diet contains 15.9% of crude protein, 3.0% of ether extract, 12% of crude fiber, 7.5% of ash, and 90% of dry matter. Feed was given at 8:00 and 15:00 h, and experimental diets were offered for 42 days. Lambs had *ad libitum* access to feed and water. Specifically, 100 g of commercial diet per Kg served was added to the previous day’s dry matter intake to ensure 10% feed refusal to secure an *ad libitum* intake. The lambs were transported 10 Km. to a commercial abattoir at the end of the feeding period (day 42). Following slaughter, the rumen contents (50 mL) were collected directly from the cranial, ventral, and caudal areas (composite sample). Afterward, the composite sample of each lamb was transported to the Lab for DNA extraction.

### 2.4. Ruminal Liquor Volatile Fatty Acids (VFAs)

Samples of the ruminal liquid were taken through an esophageal probe; a suction was generated using a vacuum pump, and 5 mL was obtained from each individual. Once the ruminal liquid was obtained, it was centrifuged at 3500 rpm for 15 min to separate the solids by density. After this, 1 mL of each sample was extracted and deposited in Eppendorf tubes, and 0.5 mL of previously prepared metaphosphoric acid was added, frozen, and stored for later analysis. The samples were thawed and centrifuged at 4300 rpm for 30 min; 500 µL of the sample supernatant was collected, making a 1:1 dilution and adding 50 µL of acetone as the internal standard. A chromatograph with a flame ionization detector was used, with an HP- INNOWAX capillary column of 30 m × 0.320 mm × 0.25. The temperature conditions were 50 °C initially for 5 min and 225 °C for 10 min, with a gradient of 5 °C per minute. The carrier gas was nitrogen, with a flow rate of 1 mL/min. To identify the individual VFAs in the sample, the retention times of acetic acid, propionic acid, and butyric acid were determined. Calculation of the concentration of individual VFAs in the sample was performed through calibration curves using serial dilutions of acetic, propionic, and butyric acid.

### 2.5. DNA Extraction and 16S rRNA Gene V3 Amplicon Sequencing

One milliliter of every rumen sample was centrifugated at 13,000 rpm, and the remaining precipitate was used for DNA extraction using the i-genomic Stool DNA Extraction Mini Kit (iNtRON Biotechnology, Inc., Seongnam-si, Republic of Korea) according to the manufacturer’s instructions. DNA was eluted in 50 μL of elution buffer, and the DNA quality and quantity were checked by agarose gel electrophoresis and a Nanodrop spectrophotometer, respectively.

Variable region 3 (V3) from the prokaryote 16S rRNA gene was amplified from the ruminal genomic DNA by polymerase chain reaction (PCR) using the V3-338f/V3-533r primers (5′-ACTCCTACGG-GAGGCAGCAG-3′/5′-TTACCGCGGCTGCTGGCAC-3′) and associated published method [21] using Phusion DNA polymerase (ThermoFisher Scientific, Waltham, Massachusetts, USA). According to the manufacturer’s instructions, the Illumina MiniSeq system generated DNA sequence data from the PCR fragments (150 × 2 cycles, paired-end sequencing). The generated MiniSeq fastq files have been submitted to the NCBI Sequence Read Archive (Accession no. PRJNA1163437).

### 2.6. Statistical Analysis

The data were analyzed using a completely randomized design (CRD) with four dietary treatments and three replicates per treatment. A general linear model (GLM) was employed to evaluate the effects of the dietary treatments on the response variables such as the volatile fatty acid concentrations and microbial composition. The mathematical model used was as follows:

Yij = μ + Ti + εij

where Yij = the observed response variable for the j-th replicate within the i-th treatment; μ = the overall mean; Ti = the fixed effect of the i-th treatment (i = 1, 2, 3, 4); and εij = the random error associated with the j-th replicate within the i-th treatment, assumed to be normally distributed (εij~N(0, σ2)). To compare treatment means, Tukey’s honest significant difference (HSD) test was performed for post hoc multiple comparisons at a significance level of α = 0.05. Statistical analyses were conducted using SAS software (version 9.0).

### 2.7. Bioinformatics Analysis

All the paired-end (PE) Illumina raw sequences were processed in R (version 4.3.2) using the DADA2 (version 1.30.0) pipeline as described by Pinteus et al. [2]. Paired-end fastq files were demultiplexed, and quality checks of forward and reverse reads were conducted based on the quality scores. Then, sequence reads were quality filtered, trimmed, and dereplicated, merging the forward and reverse reads to obtain the complete denoised sequences. The reads were inspected for removed chimeras, and amplicon sequence variants (ASVs)/operational taxonomic units (OTUs) were generated. Taxonomic classification of 16S rRNA amplicon sequence data was performed using a naive Bayes classifier (pre-trained on the SILVA 16S rRNA reference database (release 138.1), clustered at 80% similarity) with the function-assigned taxonomy.

The vegan package was used to calculate three indices of alpha diversity: Pielou evenness, richness (observed number of OTUs), and the Shannon diversity index. A nonparametric statistical test (Kruskal–Wallis test) was used to determine whether α-diversity differed among the treatment groups. Conversely, bacterial community analyses were also conducted using vegan, and the results were then visualized using “ggplot2” [3]. Beta diversity was assessed as the principal coordinate analysis (PCoA) based on Bray–Curtis distances to evaluate the community composition differences between samples. Data were visualized using principal coordinate analysis plots generated using ggplot2 within R version 4.3.2.

## 3. Results

### 3.1. Volatile Organic Compounds (VOCs) Present in Brown, Red, and Lettuce Algae by CG-MS

**Figure 1:**
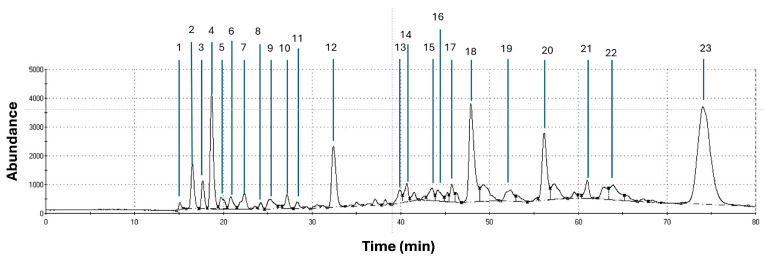
Chromatogram of total ion of the volatile compounds in Red algae by CG-MS: 1. 2-Methylbutane; 2. Diethyl ether; 3. 1,1-Dichloroethene; 4. Propanon-2-one; 5. Ethene, 1,2-dichloro-, (E)-; 6. 1-Propanol; 7. 2-butanol; 8. Benzene; 9. 1,2-Dichloroethane; 10. Pental-2-ol; 11. Octane; 12. Ethyl isovalerate; 13. 2-Isopropyl-3-methoxypyrazine; 14. p-menthatriene; 15. etenyl-dimethylpyrazine; 16. 3-nonenal; 17. ethyl 3-(methylthio)propanoate; 18. Limonene oxide; 19. Anethole; 20. Tetradecane; 21. Carbamothioic acid; 22. beta-Himachalene; 23. 2-methyl-1,4-naphthalenedione.

**Figure 2:**
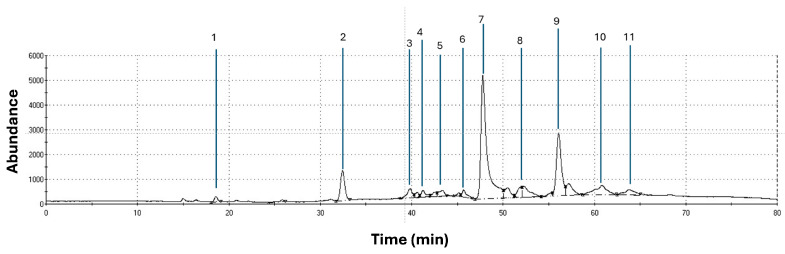
Chromatogram of total ion of the volatile compounds in Lettuce algae by CG-MS: 1. Diethyl ether; 2. Ethyl isovalerate; 3. Acetilpyrazine; 4. ethenyl-dimethylpirazine; 5. 1-2-Cyclopentanedione, 3,4; 6. Methyl salicylate; 7. Nerol; 8. trans-2-Undecenal; 9. beta-ionone; 10. 1,4-Naftalenedion; 11. Clorotalonil.

**Figure 3:**
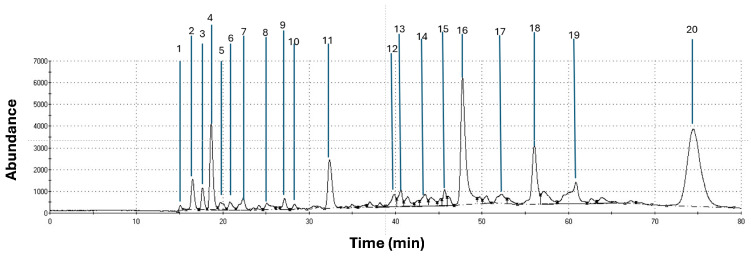
Chromatogram of total ion of the volatile compounds in Brown algae by CG-MS: 1. Butane; 2. Ethanol; 3. propanona-2-one; 4. Diethyl ether; 5. 1-Propanol; 6. Carbon disulfide; 7. butan-2-one; 8. Tri-chloroethane; 9. 1,2-Dichloropropane; 10. Methyl butanoate; 11. Octane; 12. 1-Heptanol; 13. Al-pha-Phellandrene; 14. Butylbenzene; 15. p-Menthatriene; 16. 4-Ethylphenol; 17. Anethole; 18. trans-2-undecenal; 19. delta-decalactone; 20. Octadecane.

Figure 1, Figure 2 and Figure 3 show the graphic chromatogram and chemical composition of Red, Brown, and Lettuce algae. The relevance index (Kovat’s) database identified 23, 20, and 11 compounds with significant relevance to Red, Brown, and Lettuce algae, including alcohols, aromas, phenolics, and aldehydes, some with nutraceutical properties.

### 3.2. Volatile Fatty Acids (VFAs)

The analysis of VFA showed in Table 1 that Lettuce algae increased (*p* < 0.05) the total amount of ruminal VFA in lambs supplemented with Lettuce but decreased (*p* < 0.05) in Red algae to the other treatments. The acetate concentration was reduced (*p* < 0.05) in all algae treatments. In contrast, the propionate amounts were greater (*p* < 0.05) in the lambs that received the algae treatments than in the control treatment. The algae supplementation did not affect the butyrate concentration (*p* < 0.05).

### 3.3. Rumen Microbiome

**Figure 4:**
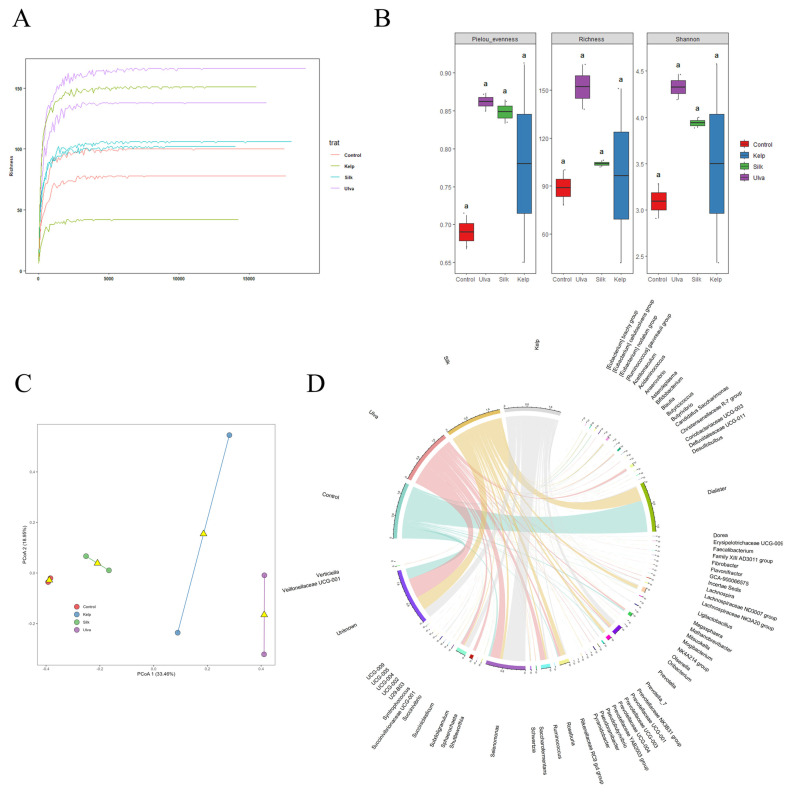
(**A**) Rarefaction curves of the four feeding treatments in lambs. (**B**) Box and whisker plots of three α-diversity indices (Pielou evenness, Richness, and Shannon diversity index) of bacterial communities in each treatment. Different letters above the whiskers denote significant differences between groups determined by Kruskal–Wallis tests (*p* < 0.05). (**C**) Nonmetric multidimensional scaling (NMDS) of bacterial communities, clustering based on Bray–Curtis similarities. (**D**) Relative abundances of bacterial genera in microbial composition among lambs fed with different diets.

We analyzed the composition of the bacterial communities after the lambs were fed four different diets using 16S rRNA gene amplicon sequencing. The amplicon sequencing generated 252,443 total reads, averaging 31,555 ± 4844 reads per sample. This was reduced to 24,169 ± 2496 when sequences were merged and quality filtered. The average number of counts per sample assigned to an OTU (post-filtering) was 12,183 ± 1817. All the samples reached the plateau phase (Figure 4A), consistent with the rarefaction curves of the bacterial population at the genus taxonomic level so that any additional increase in the number of sequences would not affect the number of genera discovered.

**Figure 5:**
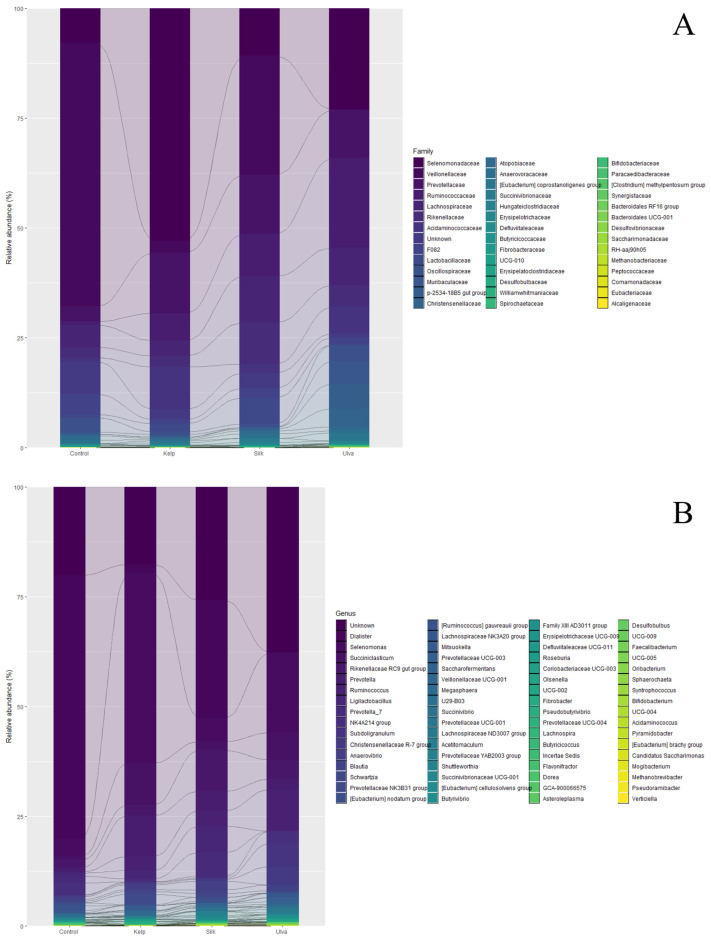
Bacterial community composition at family (**A**) and genus (**B**) levels in the rumen of four feed treatments in lambs.

The bacterial community composition in the rumen is represented in Figure 5. The most abundant species were *Firmicutes* (77.8% of relative abundance), *Bacteroidetes* (20.7% of relative abundance), and *Actinobacteriota* (0.7% of relative abundance). Within the *Firmicutes*, *Selenomonadaceae* and *Veillonellaceae* (predominant genus: *Selenomonas* and *Dialister*, respectively) dominated the rumen. The predominant families of the *Bacteroidetes* were *Prevotellaceae* (predominant genus: *Prevotella*) and *Rikenellaceae*, whereas the *Actinobacteriota* mainly consisted of the family *Atopobiaceae* (Figure 5A).

### 3.4. Microbial Diversity

Dietary macroalgae amendments did not affect the diversity of active rumen bacteria in ewes. The bacterial species richness and evenness were similar between treatments (Figure 4B). Likewise, Shannon’s diversity index showed a similar trend (Figure 4B). However, a comparison of the microbial communities within each treatment suggested that the microbial communities differed (Figure 4C). Principal coordinate analysis (PCoA) visualized the difference between the microbial communities. The communities associated with the macroalgae treatments and control differed (Figure 4C), with the first two axes of the PCoA plot explaining 52.41% of the total observed variation.

Firmicutes were our study’s most dominant bacterial phylum, being more abundant in the control than in macroalgae-fed lambs. A similar trend was observed for the dominant *Negativicutes* class, *Veillonellaceae* family, and the dominant genus of this family, *Dialister* (Figure 5B, showing a significant decrease in relative abundance, accounting only for 0.1% in Lettuce, 0.2% in Brown and 26% in Red, compared to the control (60%) (Figure 5B). On the contrary, the second most abundant genera, *Selenomonas,* showed opposite trends (Figure 4D), increasing to 18.1% in Lettuce, 43.1% in Brown, and 5.3% in Red; meanwhile, the control was 4% (Figure 5B). At the species level, *Dialister succinatiphilus* was the most highly represented taxon in the control (60%) and Red (26.6%) treatments, followed by *Selenomonas ruminantium*, with a higher relative abundance in Lettuce (18.1%) and Brown (42.1%) (Table 2).

*Bacteroidetes* was the second most abundant phylum in our study, showing changes in relative composition during different feed treatments at lower taxonomic levels, where the *Prevotellaceae* family (Figure 5A) and its representative *Rikenellaceae* RC9 gut group genus were relatively more abundant in Lettuce 4.4%, Red 9.2%, and Brown 2.4%, meanwhile, the control was 1.7%. At the species level, *Prevotella ruminicola* represented 0.19% in the control treatment and 0.29% in the Brown treatment (Table 1). *Prevotella albensis* had an increased relative abundance in Red (5.1%) and Brown (0.65%) compared to the control (0%). Interestingly, many unclassified sequences at the species level belong to the *Prevotellaceae* family (Table 1).

## 4. Discussion

Previous studies have explored the potential role of macroalgae in ruminant diets [10,20]. However, there is a significant gap in the literature regarding the combined effects of different types of macroalgae on feed utilization and rumen fermentation in ruminants [9]. While it is known that the chemical composition of macroalgae varies depending on the species, production systems, growing conditions, and harvesting methods [6], the specific implications of these variations remain poorly understood. As a result, the following discussion is based on the theoretical insights and extrapolations from existing data.

Three of the twenty-three VOCs observed in Red algae have been linked with bacterial modulation activity (Anethole, beta-Himachalene, 2-methyl-1,4-naphthalenedione) [22,23,24]. The combination of these compounds might act synergistically to modify the rumen microbiota, enhancing the production of VFAs through better carbohydrate digestion or favoring specific microbial communities that produce more VFAs [25]. Since Red algae contain various bioactive compounds, their complex effects on the rumen fermentation process can contribute to enhanced nutrient utilization, increasing VFAs [25,26].

At the same time, four of the twenty VOCs detected in Brown algae (Ethanol, Alpha-Phellandrene, 4-Ethylphenol, Anethole), and four of eleven VOCs identified in the Lettuce algae (Methyl salicylate, Nerol, 1,4-Naftalenedion, Clorotalonil) have been reported with potential to influence bacterial regulation [27,28,29]. Specifically, all these VOCs share diverse potential applications in the rumen as modulators of fermentation [28], antioxidants [23,24], antimicrobials [27], or fungicides [23,28]; thus, the few studies reported have concluded that the moment of use could focus on specific periods of livestock production [20].

In the specific case of anethole, this VOC is reported to affect the gut microbiota, including *Firmicutes*, by modulating microbial populations through its antimicrobial properties [28]. Firmicutes are a dominant bacterial phylum in the rumen, and the antimicrobial effects of these VOCs might influence their abundance and activity, as we observed in the lambs that received a source of macroalgae [29]. Therefore, the decrease in the *Veillonellaceae* family was detected in the anethole antimicrobial properties. While no specific study directly links anethole to *Veillonellaceae* regulation, its ability to modulate microbial populations suggests it could affect their abundance and activity [29]. Moreover, specific direct relationships between compounds such as beta-Himachalene, 4-Ethylphenol, and Firmicutes in the rumen have not been widely reported [30]. The overall impact of these compounds would depend on the balance between antimicrobial action and microbial adaptation in the rumen environment [28].

Although little information exists on the correlations between VOCs in the macroalgae and specific genera of bacteria in the rumen, the VOCs identified in Brown algae have diverse bioactivities that might indirectly affect *Selenomonas* populations and rumen microbiota [31]. *Selenomonas*, a key propionate-producing bacterium, thrives in anaerobic environments. Modulating microbial populations through these compounds could reduce competition, enhancing the conditions for *Selenomonas* growth, especially by influencing lactic acid and fermentation pathways [14]. Salem et al. [32] describe diverse plant bioactive as causing a positive effect, but dietary and animal factors may also modulate effects. Alpha-Phellandrene is a naturally occurring monoterpene found in the essential oils of various plants like eucalyptus and certain citrus species. In the rumen, it might act as an antimicrobial agent, potentially influencing the balance of microbial populations. Its specific impact on *Selenomonas* would depend on the compound’s concentration and interaction with other microorganisms such as *Dialister*. In some cases, alpha-phellandrene could reduce competition from different bacteria, indirectly promoting the growth of propionate-producing species like *Selenomonas* by improving fermentation conditions in the rumen or associated with other biocompounds presented in the diet as phenolics or essential oils [14].

One of these phenolic compounds described in diverse reviews is 4-Ethylphenol, which has been related as a potentiator of the fermentation by microbes in the rumen [29]. It is often associated with undesirable flavors in fermented products, like alcohol, but it may influence microbial activity in the rumen [33]. The 4-Ethylphenol VOC might modify the rumen’s microbial balance, potentially inhibiting or promoting the growth of specific bacteria [29]. Its interaction with microbes like *Selenomonas* could depend on how it impacts fermentation pathways, particularly those involved in energy production or short-chain fatty acid synthesis. Additionally, the *Prevotellaceae* family increased their population by including macroalgae in the alimentary bolus, contrasting with the control treatment. The *Prevotellaceae* family is a significant group of bacteria in the rumen, playing a crucial role in carbohydrate and protein digestion [33]. This family is known for breaking down complex carbohydrates, like starches and hemicellulose, into short-chain fatty acids, such as acetate, propionate, and butyrate, critical for ruminant energy metabolism [28]. O’Hara et al. [34] explored the impact of red seaweeds like *Asparagopsis* taxiformis on the rumen microbial ecosystem. The study found that *Asparagopsis* supplementation effectively reduced methane production [3,34]. Still, it also altered the overall microbial community, including *Prevotellaceae*, which is essential for fiber degradation and volatile fatty acid production. The presence of bioactive compounds in these algae, like bromoform, is believed to be responsible for these microbial shifts, potentially affecting nutrient metabolism and efficiency in ruminants. In the few studies reported using the same macroalgae, Lee-Rangel et al. [20] observed that *Mazzaella* spp. reduced the emission of CO_2_ in an in vitro gas study compared with *Macrocystis pyrifera* and *Ulva* spp.; also, the authors reported that the three algae modulate the cumulative in vitro gas production in different ways. Additionally, Du et al. [35] described how changes in feed composition, including the introduction of macroalgae or other dietary changes, can affect ruminal communities. Specifically, shifts in diet impact the abundance of *Prevotella*, a genus closely associated with carbohydrate metabolism and protein degradation in the rumen. These dietary adjustments could enhance the digestion and absorption of nutrients, contributing to livestock growth and productivity.

Finally, our study joins the few reports that characterize the applications and microbiota modulation in the rumen by using macroalgae. Despite these positive outcomes, we identified similar limitations as described by Cheong et al. [30], such as the lack of long-term studies and the variability in the responses among different ruminants, specifically using diets that include macroalgae, with analysis of the animals’ performance in various periods, such as the fattening phase, lactating period, or gestational phase. To address these shortcomings, we recommend conducting more comprehensive, long-term studies. One of them could explore the performance of sheep production during the fattening lamb phase using a similar dose to our study, evaluating the energy saved by the VOCs presented in our research and the theorization described previously. In addition, when assessing the effectiveness of these feed additives, we must consider their practical implementation and feasibility. Factors such as cost-effectiveness, scalability, types of macroalgae, dosage, supplementation duration, and performance should be carefully evaluated to determine the viability of incorporating macroalgae, such as Lettuce, Brown, or Red, into livestock production systems. Furthermore, long-term studies and comprehensive animal health assessments are essential to ensure the safety and well-being of ruminant livestock.

## 5. Conclusions

This study contributes to the new evidence supporting the use of macroalgae in ruminant diets to modulate rumen microbial populations and enhance feed utilization. The diversity in chemical composition among macroalgae species, such as Red, Brown, and Lettuce, introduces a range of bioactive compounds, particularly VOCs like anethole, beta-himachalene, and 4-ethylphenol, which demonstrate antimicrobial and fermentation-modulating properties. These VOCs influence vital bacterial groups, such as *Firmicutes*, *Veillonellaceae*, and *Prevotellaceae*, all crucial for carbohydrate and protein digestion in the rumen.

Modifying these microbial populations, notably the promotion of *Selenomonas* and the shifts in *Prevotella* abundance suggests that incorporating macroalgae into diets could improve fermentation efficiency and overall animal performance. However, the observed microbial shifts, particularly in methane-reducing bacteria, point to long-term studies considering the impacts on microbial diversity and ruminant health. Furthermore, the practical challenges of implementing macroalgae in large-scale feeding systems, such as cost, scalability, and dosage optimization, require careful evaluation. Future research should focus on extended trials across different livestock production phases to fully assess the potential of macroalgae to enhance productivity while maintaining the health and well-being of the animals.

## Figures and Tables

**Table 1:** Fermentation pattern of lambs supplemented with *Ulva* spp., *Mazzaella* spp., and *Macrocystis pyrifera*.

	Control	*Ulva* spp.	*Mazzaella* spp.	*Macrocystis pyrifera*	SEM ^1^	*p* Value
Totales VFA, mol/L	116 ^b^	240.6 ^c^	75.8 ^a^	101.9 ^b^	8.32	<0.01
Acetate, mol/100 mol	44.8 ^a^	35.9 ^b^	36.1 ^b^	37.1 ^b^	3.52	0.02
Propionate, mol/100 mol	43.5 ^b^	51.1 ^b^	52.4 ^b^	50.1 ^b^	4.69	0.01
Butyrate, mol/100 mol	11.5	12.9	11.5	12.6	0.83	0.09

^1^ Standard error of the mean. ^abc^ Means within a row with different superscripts differ (*p* < 0.05).

**Table 2:** Relative abundances (%) at the species level in the rumen of four feed treatments in lambs.

Family	Species	Control	Lettuce	Red	Brown
Veillonellaceae	*Dialister succinatiphilus*	60.0	0.1448	26.6	2.0
Prevotellaceae	Unknown	34.4	80.9	59.7	50.5
Selenomonadaceae	*Selenomonas ruminantium*	3.9	18.1	4.1	42.1
Selenomonadaceae	*Anaerovibrio lipolyticus*	0.6187	0.0462	1.4	1.0
Prevotellaceae	*Prevotella ruminicola*	0.1987	0	0	0.2919
Oscillospiraceae	*Flavonifractor plautii*	0.1792	0	0	0
Prevotellaceae	*Prevotella bryantii*	0.1650	0	1.6	0
Veillonellaceae	*Megasphaera elsdenii*	0.1277	0	0.2207	0.2609
Selenomonadaceae	*Mitsuokella multacida*	0.1132	0	0	0.4348
Lachnospiraceae	*Eubacterium cellulosolvens*	0.1104	0	0.4771	0.0902
Acidaminococcaceae	*Acidaminococcus fermentans*	0.0483	0	0.0332	0
Fibrobacteraceae	*Fibrobacter succinogenes*	0.0170	0	0	0.2802
Methanobacteriaceae	*Methanobrevibacter millerae*	0.0142	0	0	0
Prevotellaceae	*Prevotella albensis*	0	0	5.1	0.6571
Ruminococcaceae	*Ruminococcus albus*	0	0	0	0.2899
Erysipelatoclostridiaceae	*Asteroleplasma anaerobium*	0	0	0.0142	0
Bifidobacteriaceae	*Bifidobacterium animalis*	0	0.0733	0	0
Ruminococcaceae	*Ruminococcus callidus*	0	0	0.2413	0
Prevotellaceae	*Prevotella corporis*	0	0	0.1068	0.2287
Ruminococcaceae	*Ruminococcus flavefaciens*	0	0	0.0534	0.8955
Veillonellaceae	*Megasphaera hexanoica*	0	0	0	0.3704
Lachnospiraceae	*Roseburia hominis*	0	0	0	0.3704
Acidaminococcaceae	*Succiniclasticum ruminis*	0	0.2157	0	0
Bifidobacteriaceae	*Bifidobacterium thermophilum*	0	0	0.0249	0
Atopobiaceae	*Olsenella umbonata*	0	0.3669	0	0

## Data Availability

Data are contained within the article.
